# Retraction: “MicroRNA‐219 decreases hippocampal long‐term potentiation inhibition and hippocampal neuronal cell apoptosis in type 2 diabetes mellitus mice by suppressing the NMDAR signaling pathway,” by Zhang, L, Chen, Z‐W, Yang, S‐F, et al. CNS Neurosci Ther. 2019; 25: 69–77

**DOI:** 10.1111/cns.13709

**Published:** 2021-07-30

The above article, first published online on May 27, 2018, in Wiley Online Library (https://onlinelibrary.wiley.com/doi/full/10.1111/cns.12981), has been retracted as requested by the journal's editorial team and with the agreement of John Wiley & Sons Ltd. The retraction has been agreed following a formal investigation into the allegation of potential scientific misconduct, which revealed that several image elements of the experimental data were published elsewhere in a different scientific context or repeatedly used within the images. Thus, the editors consider the conclusions of this article to be invalid. The authors were not available to comment on these inconsistencies despite of multiple inquires by the journal.

